# ﻿*Melicopeiolensis* (Rutaceae), a new tree species from Kaua‘i, Hawaiian Islands

**DOI:** 10.3897/phytokeys.250.128963

**Published:** 2024-12-31

**Authors:** Kenneth R. Wood, David H. Lorence, Warren L. Wagner, Marc S. Appelhans

**Affiliations:** 1 National Tropical Botanical Garden, 3530 Papalina Road, Kalāheo, HI 96741, USA National Tropical Botanical Garden Kalāheo United States of America; 2 National Museum of Natural History, Smithsonian Institution, PO Box 37012, Washington, DC 20013-7012, USA National Museum of Natural History, Smithsonian Institution Washington United States of America; 3 Department of Systematic Botany, Albrecht-von-Haller Institute of Plant Sciences, University of Göttingen, Untere Karspüle 2, 37073 Göttingen, Germany University of Göttingen Göttingen Germany

**Keywords:** Conservation, discovery, endangered tree species, Hawaiian flora, *Melicope* section *Pelea*, Sapindales, single island endemism

## Abstract

A newly-discovered endemic tree species of *Melicope* from Kaua‘i, Hawaiian Islands, is described and illustrated with notes on its distribution, ecology, conservation status and phylogenetic placement. A modification to the existing key to Hawaiian *Melicope* is also provided. *Melicopeiolensis***sp. nov.** is a member of Stone’s *Megacarpa* group having carpels connate at base, capsules 4-lobed and leaves usually opposite. The new species differs from its Hawaiian congeners by its unique combination of abaxially glabrate to pilose-pubescent leaves with petioles up to 70 mm long, ramiflorous and axillary inflorescences, sepals on staminate flowers 0.3–0.5 mm long, capsules with green and purple streaking, 10–14 mm wide and seeds 3–3.5 mm long. Since its discovery in 2021, 15 individuals have been documented within a single remote windward hanging valley below the Kawaikini Summit of Kaua‘i. *Melicopeiolensis* represents a new Critically Endangered (CR) single island endemic species in need of conservation.

## ﻿Introduction

*Melicope* J.R.Forst. & G.Forst. is the largest genus within Rutaceae (Citrus family) containing ca. 239 species of shrubs and trees distributed across the Malagasy and Indo-Himalayan regions, SE Asia, Australasia and the Pacific Islands (Hartley 2001; Appelhans et al. 2014b, 2017; Wood et al. 2016, 2017). The Hawaiian lineage was initially placed in the genus *Pelea* A.Gray, but both morphological (Hartley and Stone 1989; Hartley 2001) and molecular phylogenetic studies (Harbaugh et al. 2009; Appelhans et al. 2014a) confirm that *Pelea* is nested within the genus *Melicope*.

Hartley (2001) recognised four sections in *Melicope* and placed the Hawaiian lineage in sect.Pelea (A.Gray) Hook.f. along with species distributed from Taiwan, Southeast Asia and Marquesas Islands to New Caledonia. Subsequently, Appelhans et al. (2017) merged the Hawaiian endemic genus *Platydesma* H.Mann into M.sect.Pelea and excluded the New Caledonian species from that section in order to preserve its monophyly. They also suggested the four sections of *Pelea* proposed by Stone (1969) and the former genus *Platydesma* be called subsections of M.sect.Pelea, but no combinations were made as they were waiting for a better understanding of relationships with Hawaiian *Melicope* (Appelhans et al. 2017). Botanists still use Stone’s species groups (sections of *Pelea*) for helping to key out the Hawaiian taxa: Apocarpa with carpels distinct in fruit, leaves opposite; *Pelea* with carpels connate at base, leaves in whorls; Megacarpa with carpels connate at base, capsules 4-lobed, leaves usually opposite; and Cubicarpa with capsules cuboid, leaves opposite (Wagner et al. 1999). However, the species groups are in need of revision since only *Pelea* was resolved as monophyletic (Appelhans et al. 2014a; Paetzold et al. 2019).

Phylogenetic analysis reveals that the Hawaiian lineage was the result of a single long-distance colonisation event, originating from an Australasian ancestor with diversification dating back ca. 7.5 mya to the Late Miocene or Early Pliocene (Appelhans et al. 2018; Paetzold et al. 2019) and pre-dating the oldest current high island of Kaua‘i (i.e. ca. 5 mya, Price and Clague (2002)). *Melicope* rely on birds for long-distance dispersal (Hartley 2001). Despite the isolated position of the Hawaiian Islands, the archipelago is not a dead-end of dispersal for *Melicope* as two independent colonisation events from the Hawaiian to the Marquesas Islands occurred in the genus (Appelhans et al. 2014a, 2018; Paetzold et al. 2019).

Hawaiian *Melicope* are one of the four most species rich plant radiations in the archipelago, having undergone extraordinary morphological and ecological diversification with 54 accepted endemic species (Wagner et al. 1999; Wood et al. 2016, 2017; Appelhans et al. 2017). Many occur throughout dry, mesic and wet forest habitats, including bogs and cliffs and range from 300–1700(–2060) m elevation. Kaua‘i holds the greatest diversity of *Melicope* with 22 species, 16 of which are single island endemics (SIE). Unfortunately, there are now 22 Hawaiian *Melicope* species that are federally listed as endangered and four considered possibly extinct (Wood 2011; Wood et al. 2016, 2019). Having limited land mass, tropical islands are particularly vulnerable to human disturbance, especially the impact of introduced non-native plant and animal species which can quickly spread and degrade remote natural ecosystems.

With Hawaii State, federal and non-government agencies conducting research and protecting the biotic diversity of Kaua‘i, SIE continue to be discovered. The > 1900 *Melicope* collections at PTBG represent a long intensive focus on the genus. The collection includes five recently discovered and described *Melicope* species from Hawaiian, Marquesas and Austral Islands. This collection has helped to guide conservationists with data on the distribution and abundance of rare Pacific Island species and also houses collections representing rediscoveries of species previously thought extinct, including nine Hawaiian *Melicope* (Appelhans et al. 2014b; Wood et al. 2016, 2017; Lorence and Wagner 2020; Wood and Walsh 2022).

**Figure 1.**
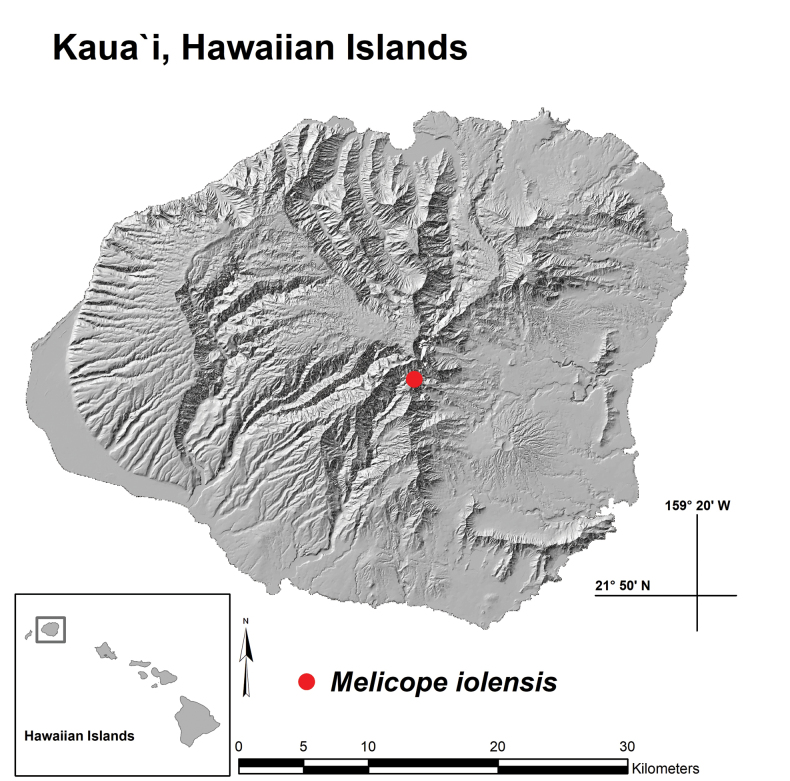
Distribution map (Kaua‘i, Hawaiian Islands) with red dot indicating single known location of *Melicopeiolensis* K.R.Wood, Lorence & W.L.Wagner in ‘Iole Valley.

In October of 2021, the lead author and members of the Kaua‘i Plant Extinction Prevention Program (PEPP) documented an unusual tree species with exceedingly small 4-lobed capsules (Megacarpa) in a remote isolated hanging valley below the central summit peak of Kawaikini (Fig. 1). Further exploration and research subsequently revealed that it differed from all other known *Melicope* species by its unique combination of abaxially glabrate to pilose-pubescent leaves with petioles up to 70 mm long, ramiflorous and axillary inflorescences, sepals on staminate flowers 0.3–0.5 mm long, capsules with green and purple streaking, 10–14 mm wide and seeds 3–3.5 mm long (Table 1). We hereby describe and name this new species *Melicopeiolensis* K.R.Wood, Lorence & W.L.Wagner, present a summary of its distribution and ecology, provide a diagnostic key with distinguishing characters, evaluate its phylogenetic position, present a table comparing it to other Kaua‘i members of Megacarpa and provide a preliminary conservation assessment using IUCN Red List criteria. This publication brings the number of recognised *Melicope* species in the Hawaiian Islands to 55 and attests to the remarkable floristic diversity of Kaua‘i, exceeding all other Hawaiian Islands with its total of 254 SIE vascular plant taxa.

## ﻿Methods

Research in the type locality has been conducted from 1994 to present. All morphological measurements were taken from dried herbarium specimens and field notes and are presented in the descriptions as follows: length × width, followed by units of measurements (mm, cm or m). The authors have examined all specimens cited and have worked extensively with *Melicope* specimens at BISH, PTBG and US. We assessed the extinction risk for *Melicopeiolensis* following the IUCN Red List Categories and Criteria (IUCN 2012, 2022). The extent of occurrence (EOO) and area of occupancy (AOO) were calculated by using ArcMap 10.6.1 in relation to coordinates recorded while collecting herbarium specimens or making field observations. Lat/Long coordinates have been truncated to protect exact locations from unauthorised access.

## ﻿Taxonomic treatment

### Melicope iolensis

Taxon classificationPlantaeSapindalesRutaceae

K.R.Wood, Lorence & W.L.Wagner sp. nov.

13DD2C10-8B4F-5207-926E-BD660C35C89F

urn:lsid:ipni.org:names:77354456-1

**Figure 2.**
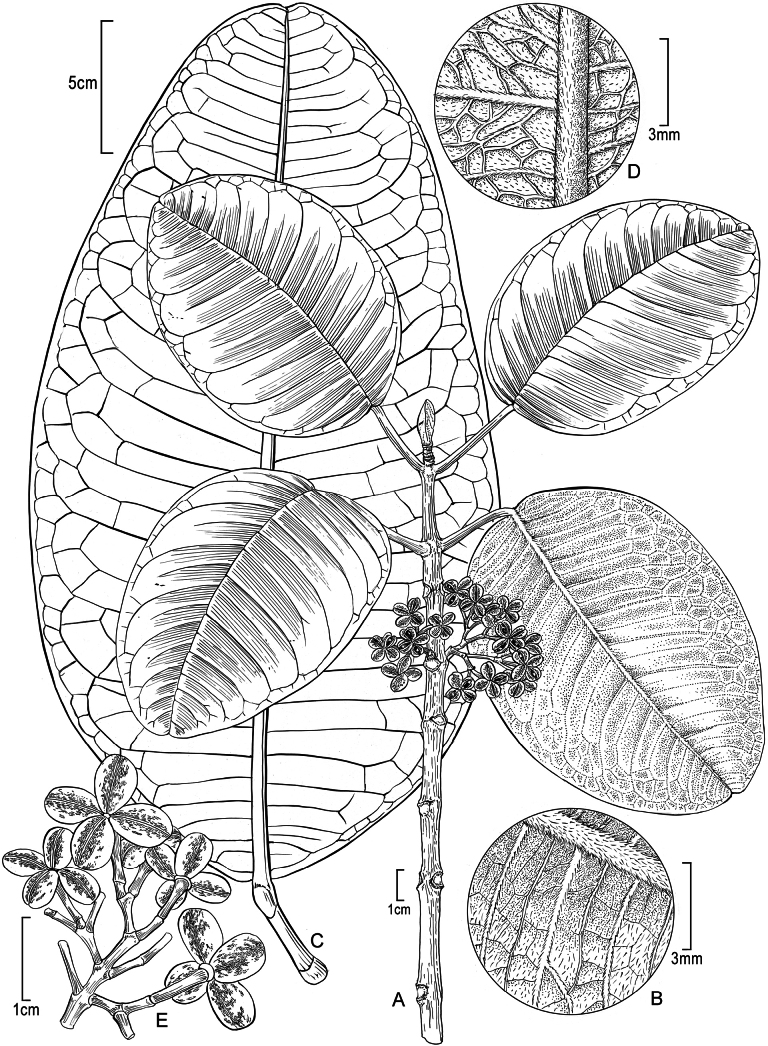
*Melicopeiolensis* K.R.Wood, Lorence & W.L.Wagner **A** fruiting branch **B** abaxial leaf surface on branch in A showing pubescence **C** abaxial surface of large leaf showing marginal and secondary venation **D** abaxial surface of large leaf in C showing scattered minute pubescence **E** portion of infructescence with connate capsules (Megacarpa) showing irregular streaking **A–E** from photos of holotype, *Wood, Heintzman & Deans 19143* (PTBG, US) (Illustration by Alice Tangerini).

**Figure 3.**
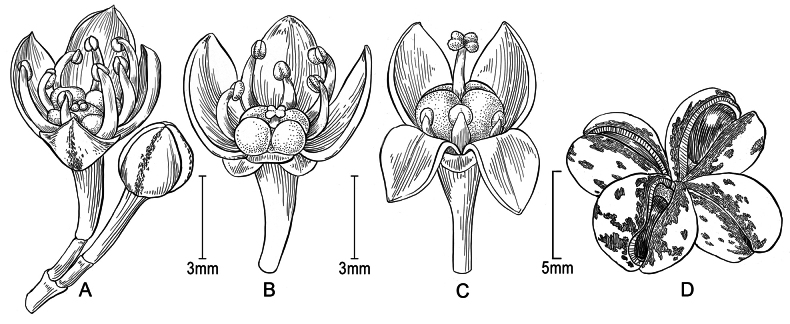
*Melicopeiolensis* K.R.Wood, Lorence & W.L.Wagner **A** staminate flower and bud **B** staminate flower, lateral view with petal cut away to show antisepalous and antipetalous stamens and two minute sepals **C** pistillate flower, lateral view with two petals folded down to show staminodes and pistil and one minute sepal **D** dehisced fruit, showing seeds **A, B** 20 Oct 2021, from photos of *Wood, Heintzman & Deans 18830* (PTBG) **C** 10 Aug 2023, from photos of *Wood, Heintzman & Deans 19369* (PTBG) **D** from holotype, 8 Sep 2023, *Wood, Heintzman & Deans 19143* (PTBG, US) (Illustration by Alice Tangerini).

**Figure 4.**
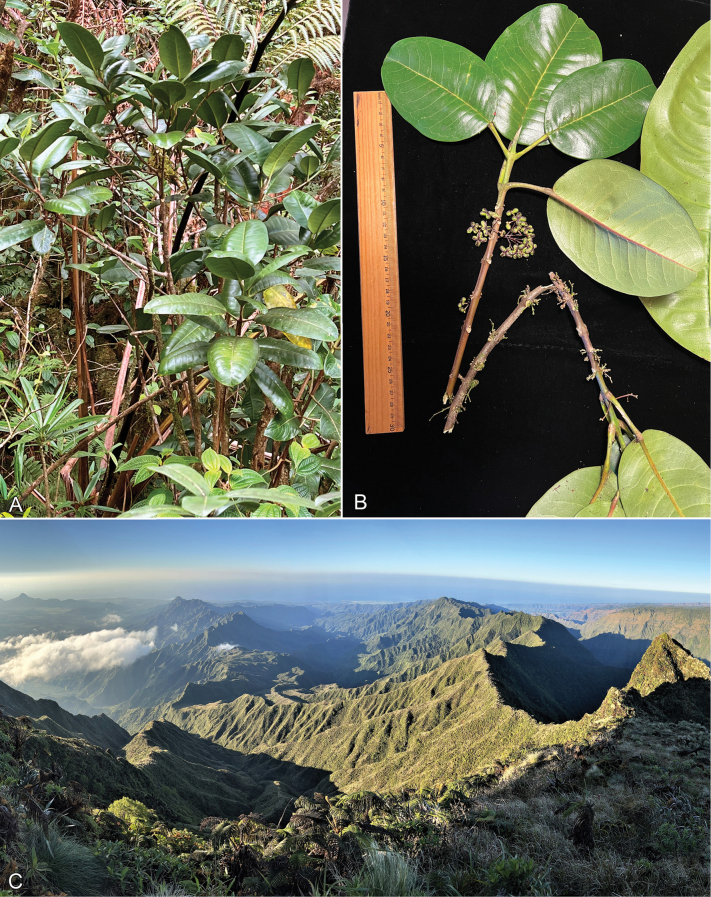
*Melicopeiolensis* K.R.Wood, Lorence & W.L.Wagner **A** habit of young tree **B** fruiting branch with axillary and fasciculate cymes **C** habitat, looking down into hanging valleys below Kawaikini summit. All photos by K.R. Wood. **A** 29 Dec 2022, *Wood & Perlman 19245* (PTBG) **B** from holotype, 8 Sep 2022, *Wood, Heintzman & Deans 19143* (PTBG, US) **C** 28 Jan 2022.

**Figure 5.**
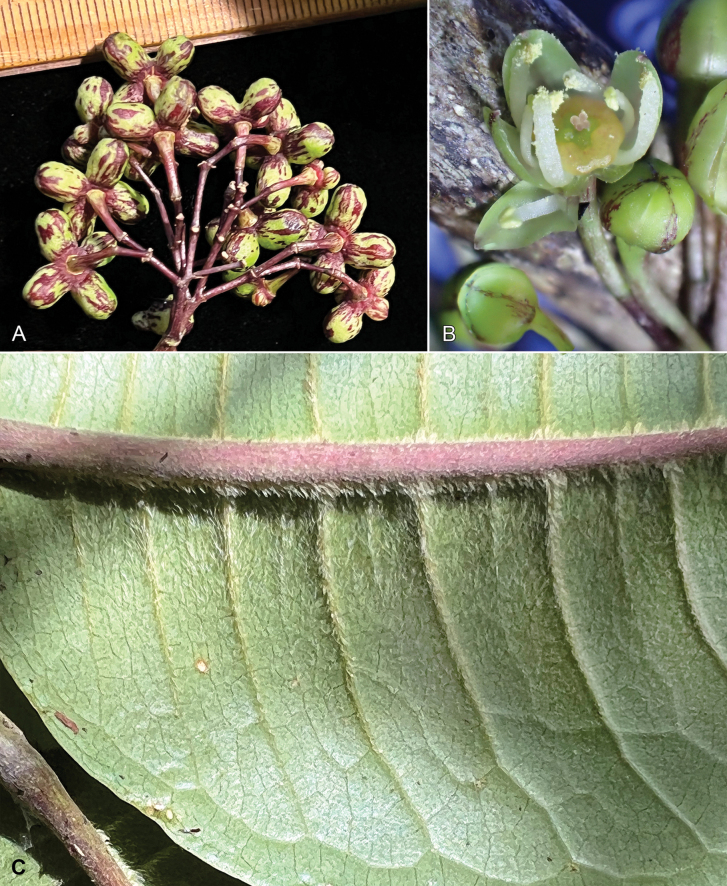
*Melicopeiolensis* K.R.Wood, Lorence & W.L.Wagner **A** infructescence with connate capsules (Megacarpa) showing green and purple streaking **B** staminate flower and buds **C** abaxial leaf surface showing close-up of pubescence. All photos by K.R. Wood. **A, C** from holotype, 8 Sep 2022, *Wood, Heintzman & Deans 19143* (PTBG, US) **B** 20 Oct 2021, *Wood, Heintzman & Deans 18830* (PTBG).

Figs 2 , 3 , 4A, B , 5

#### Diagnosis.

*Melicopeiolensis* is morphologically most similar to *M.wawraeana* (Rock) T.G.Hartley & B.C.Stone, but differs by its combination of leaves abaxially glabrate to pilose-pubescent (vs. glabrous), inflorescence ramiflorous, rarely axillary (vs. axillary), shorter sepals on staminate flowers, 0.3–0.5 mm long (vs. 3.5 mm) and smaller seeds 3–3.5 mm long (vs. 5–8 mm). Phylogenetically, *M.iolensis* is most closely related to *M.nealae* (B.C.Stone) T.G.Hartley & B.C.Stone, yet starkly differs by its tree habit (vs. shrub), flowers usually 9–18 per inflorescence (vs. 1–5), carpels with exocarp glabrous, connate 1/6–1/5 their length (vs. puberulent, connate 1/2–3/4 length) and seeds 3–3.5 mm long (vs. 5–8 mm long).

#### Type.

**USA** • **Hawaiian Islands, Kaua‘i**: Līhu‘e District, ‘Iole headwaters, ♀, 22.042, -159.497, 872 m alt., 8 Sep 2022 (fr.), *K.R. Wood, S. Heintzman & S. Deans 19143* (holotype: mounted on 2 sheets, PTBG1000096868, PTBG1000096869!; isotype (to be distributed): US!).

#### Description.

***Trees*** 3–8 m tall, trunks up to 20 cm diameter, bark smooth, mottled grey-brown, ultimate stems brown-red, new growth and young branchlets sparsely tan puberulent, glabrate in age. ***Leaves*** opposite, unifoliolate; petiole (20–)30–70 mm long, strigose-pubescent, adaxially glabrous, shallowly canaliculate; the blade subcoriaceous to coriaceous, ovate, oblong-ovate, oblong-elliptic, (8–)14–25(–33) × (6–)10–18 cm, margin entire; base truncate to obtuse, rarely acute; apex rounded, often emarginate; secondary veins 15–20 pairs, connected by an arched vein 4–20 mm from margin; higher order venation reticulate; adaxial surface glabrous; abaxial surface minutely black glandular punctate, pilose-pubescent, tan-yellow, or glabrate; mid-rib usually densely pilose-strigose; secondary veins pilose-strigose. ***Inflorescences*** in ramiflorous, densely fasciculate cymes, occasionally axillary, (4–)9–18 flowered, to 40 mm long, purple-red when fresh; peduncles 2–5 mm long, glabrate, branched to second degree; primary branches 2–4 pairs; pedicels 4–14 mm long, glabrate; bracteoles triangular-ovate 0.3–0.4 mm long. ***Flowers*** unisexual, 4-merous; perianth glabrate; androecium, nectary disc and gynoecium glabrous; ovary greenish glabrous; stigma 4-lobed ca. 0.5 mm diameter; staminate flowers with sepals orbicular-ovate, free, 0.3–0.5 × 0.5–0.7 mm, glabrate; petals green with purple streaks, narrowly deltate to ovate, 2.5–4.0 mm long; stamens 8; antisepalous filaments ca. 3.5 mm long; antipetalous filaments ca. 2.5 mm long; anthers ellipsoid ca. 0.5–0.6 mm long, with pollen; style obsolete; pistillate flowers with sepals orbicular-ovate, free, 1.0 × 1.3–1.5 mm; petals green, dorsally purple, narrowly deltate to ovate, free, 2.8–3.0 mm long; staminodes 8; antisepalous filaments ca. 0.5 mm long; antipetalous filaments ca. 0.4 mm long; anthers ovoid-ellipsoid ca. 0.5–0.6 mm long, no pollen observed; style 1.5 mm long. ***Capsules*** green with purple irregular streaking when fresh, 3–4 × 10–14 mm; carpels basally connate 1/6 to 1/5 their length, exocarp glabrous; endocarp glabrous. ***Seeds*** 1–2 per carpel, ovoid, 3–3.5 mm long.

#### Additional specimens examined (paratypes).

**USA. Hawaiian Islands, Kaua‘i**: Līhu‘e District; all collections from ‘Iole headwaters • 1 ♂; 884 m alt.; 20 Oct 2021 (fl.); *Wood et al.18830* (PTBG) • 1 ♀; 884 m alt.; 20 Oct 2021 (fr.); *Wood et al. 18831* (PTBG, US) • 1 ♂; 884 m alt.; 20 Oct 2021 (fl.); *Wood et al. 18832* (BISH, PTBG) • 1 ♀; 884 m alt.; 20 Oct 2021 (fr.); *Wood et al. 18833* (PTBG) • 1 ♀; 884 m alt.; 8 Sep 2022; *Wood et al. 19146* (PTBG, US) • 1 ♀; 900 m alt.; 8 Sep 2022 (fl.); *Wood et al. 19148* (UC, PTBG) • 914 m alt.; 8 Sep 2022; *Wood et al. 19151* (BISH, NY, PTBG, UC, US) • 890 m alt.; 29 Dec 2022; *Wood & Perlman 19245* (PTBG) • 1 ♂; 884 m alt.; 10 Aug 2023 (fl.); *Wood et al. 19367* (PTBG) • 1 ♂; 884 m alt.; 10 Aug 2023 (fr.); *Wood et al. 19368* (PTBG); • 884 m alt.; 10 Aug 2023 (fl.); *Wood et al. 19369* (PTBG) • 1 ♀; 911 m alt.; 10 Aug 2023 (fl.); *Heintzman et al. KP08102301* (PTBG).

#### Phenology.

*Melicopeiolensis* has been observed with flowers during the months of August and October and with fruit in August, September and October.

#### Etymology.

The name *Melicope* is derived from the Greek *meli*, honey and *kope*, cut in pieces, alluding to the lobed floral nectary (Lorence and Wagner 2020) and the species epithet represents the holotype locale, ‘Iole, which literally means “rat” in the Hawaiian language (Pukui et al. 1974).

#### Affinities.

Molecular phylogenetic analyses, based on RADseq datasets (Restriction-Site Associated DNA-sequencing; see Paetzold et al. (2019) for methodology), resolve *Melicopeiolensis* in a clade that includes all taxa belonging to Cubicarpa and Megacarpa and as being most closely related to *M.nealae* (Appelhans et al., in preparation). *Melicopeiolensis* can be distinguished from the latter species by its tree habit (vs. shrub); flowers usually 9–18 per inflorescence (vs. 1–5); sepals of staminate flowers 0.3–0.5 mm long (vs. 2.5 mm long); capsules with green and purple streaking, up to 14 mm wide (vs. green, up to 27 mm wide), carpels with exocarp glabrous, connate 1/6–1/5 their length, (vs. puberulent, connate 1/2–3/4 length); and seeds 3–3.5 mm long (vs. 5–8 mm long) (Table 1). *Melicopenealae* was previously thought extinct with only two known collections made in 1909 and 1960 and has long been looked for by NTBG Science staff. It was recently rediscovered in transitional mesic to wet forests of western Kaua‘i (Wood and Walsh 2022), is currently known from only 11 individuals and is being monitored and conserved by PEPP and NTBG.

Morphologically, *Melicopeiolensis* is most similar to *M.wawraeana*, but can easily be separated by its combination of leaves abaxially glabrate to pilose-pubescent (vs. glabrous on *M.wawraeana*); inflorescence ramiflorous, rarely axillary (vs. axillary); sepals on staminate flowers glabrous, 0.3–0.5 mm long (vs. puberulent, 3.5 mm long); capsules with endocarp and exocarp glabrous, connate 1/6–1/5 their width, up to 14 mm wide, with green and purple streaking (vs. endocarp and exocarp usually sparsely puberulent, connate 1/2 width, up to 20 mm wide, dark green); and seeds 3–3.5 mm long (vs. 5–8 mm) (Table 1). *Melicopewawraeana* is quite common and known from Kaua‘i and O‘ahu (Wagner et al. 1990, 1999).

*Melicopeiolensis* is not closely comparable morphologically to any of the remaining Hawaiian Megacarpa taxa. Specifically on Kaua‘i, as a tree up to 8 m tall, it differs from the shrubs *M.feddei* (H.Lév.) T.G.Hartley & B.C.Stone, *M.kavaiensis* (H.Mann) T.G.Hartley & B.C.Stone and *M.macropus* (Hillebr.) T.G.Hartley & B.C.Stone. It also differs from those three shrub species in having longer petioles and leaves, inflorescence ramiflorous, rarely axillary, shorter sepals and smaller capsules and seeds (Table 1). The only two remaining Megacarpa taxa on Kaua‘i are *M.cruciata* (A.Heller) T.G.Hartley & B.C.Stone and *M.puberula* (H.St.John) T.G.Hartley & B.C.Stone, from which *M.iolensis* also starkly differs in having longer petioles and leaves, inflorescence ramiflorous, rarely axillary, shorter sepals, glabrous endocarp and smaller capsules and seeds (Table 1).

#### Distribution and ecology.

*Melicopeiolensis* is endemic to the volcanic island of Kaua‘i (Fig. 1), where it is known from only 15 individuals located in the remote, upper headwater valley of ‘Iole. The type location is in a hanging valley, having vertical cliffs above and a series of cliffs and waterfalls below, isolating its accessibility (Fig. 4C).

The plant community where *Melicopeiolensis* occurs is a *Metrosideros* Banks ex Gaertn. (Myrtaceae) / *Cheirodendron* Nutt. ex Seem. (Araliaceae) montane wet forest with matting ferns of *Dicranopteris* Bernh. and *Diplopterygium* (Diels) Nakai (Gleicheniaceae) and a dissecting riparian drainage. The forested slopes are steep with a diverse mixture of native sedges, grasses, ferns, herbs, shrubs and trees, along with a high density of terrestrial and epiphytic bryophytes throughout. Associated genera of trees include *Polyscias* J.R.Forst. & G.Forst. (Araliaceae); *Pritchardia* Seem. & H.Wendl. (Arecaceae); *Dubautia* Gaudich. (Asteraceae); *Cyanea* Gaudich. (Campanulaceae), *Perrottetia* Kunth (Dipentodontaceae); *Antidesma* L., *Euphorbia* L. (Euphorbiaceae); *Hydrangea* Gronov. (Hydrangeaceae); *Geniostoma* J.R.Forst. & G.Forst. (Loganiaceae); *Eurya* Thunb. (Pentaphylacaceae); *Myrsine* L. (Primulaceae); *Syzygium* Gaertn. (Myrtaceae); *Bobea* Gaudich., *Coprosma* J.R.Forst. & G.Forst., *Kadua* Cham. & Schltdl., *Psychotria* L. (all Rubiaceae); *Melicope* J.R.Forst. & G.Forst. (Rutaceae); and *Pipturus* Wedd. and *Touchardia* Gaudich. (Urticaceae). Genera of sedges and grasses include *Carex* L., *Cyperus* L., *Machaerina* Vahl (Cyperaceae); *Eragrostis* Wolf, *Panicum* L. (Poaceae); herbs and shrubs include *Bidens* L. (Asteraceae); *Vaccinium* L. (Ericaceae); *Cyrtandra* J.R.Forst. & G.Forst. (Gesneriaceae); and the woody climber *Freycinetia* Gaudich. (Pandanaceae). Genera of ferns include *Asplenium* L., *Hymenasplenium* Hayata (Aspleniaceae); *Deparia* Hook. & Grev., *Diplazium* Sw. (Athyriaceae); *Sadleria* Kaulf. (Blechnaceae); *Cibotium* Kaulf. (Cibotiaceae); *Microlepia* C.Presl (Dennstaedtiaceae); *Ctenitis* (C.Chr.) C.Chr. (Dryopteridaceae); *Hoiokula* S.E.Fawc. & A.R.Sm. and *Menisciopsis* (Holttum) S.E.Fawc. & A.R.Sm. (Thelypteridaceae).

**Table 1.** Comparison of morphological characters of all eight Kaua‘i *Melicope* species with carpels connate at base, capsules 4-lobed, and leaves opposite (i.e., Megacarpa).

Character	*M.iolensis*	*M.cruciata*	*M.feddei*	*M.kavaiensis*	*M.macropus*	*M.nealae*	*M.puberula*	*M.wawraeana*
Habit	Tree	Tree	Shrub	Shrub	Shrub	Shrub	Tree	Tree
Leaf length (cm)	(8–)14–25(–33)	8–17	2–8(–14)	5.5–18	10–15	3–18	6–17	4–20(–30)
Abaxial leaf pubescence	Glabrate to pilose–pubescent	Sparsely pilose	Glabrous	Sparsely pilose	Glabrate	Pilose–pubescent	Sparsely pilose	Glabrous
Petiole length (mm)	(20–)30–70	10–35	5–25	10–40	12–20	10–30	20–30	10–50
Inflorescence	Ramiflorous and axillary	Axillary	Axillary	Axillary	Axillary	Axillary	Axillary	Axillary
# of flowers	9–18	3–6(–12)	1–5(–15)	(1–)3–9(–11)	1–3	1–5	3–9(–15)	5–15(–21)
♂ Sepal length (mm)	0.3–0.5	3–3.5	2–2.5	3.5–5.5	Unknown	2.5	3–3.5	3.5
♀ Sepal length (mm)	1.0	3.5–5	2–2.5	2–4	1.5	2.5	2–4.5	3.0
♀ Sepal indumentum	Glabrous	Puberulent	Glabrous to sparsely puberulent	Glabrate to sparsely puberulent	Minutely puberulent	Puberulent	Puberulent	Puberulent
Capsule width (mm)	10–14	24–34	16–25(–30)	(13–)18–40	25–35	20–27	14–20	11–20
Carpel length (mm)	4–6	12–17	7–12(–14)	8–20	12–18	10–12	7–10	6–7
Carpel % connate	1/6–1/5	1/4	1/6–1/4	1/5–1/2	1/6	1/2–3/4	1/2	(1/3–)1/2
Capsule color	Green w/ purple streaking	Green	Reddish green	Green	Green	Green	Dark red	Dark green
Pubescence on exocarp	Glabrous	Glabrous	Glabrous	Glabrous	Sparsely puberulent	Puberulent	Puberulent	Puberulent to glabrous
Pubescence on endocarp	Glabrous	Densely short villous	Glabrous	Glabrous	Glabrous	Glabrous	Short villous	Sparsely puberulent to glabrous
Seed length (mm)	3–3.5	7.5	4–8	6–10	5–6	5–8	5–6	5–8

### ﻿Modification of existing key to Hawaiian *Melicope* (in Wagner et al. 1990, 1999)

To accommodate *Melicopeiolensis*, the following couplets can be inserted into the existing key to Hawaiian *Melicope* (treated as *Pelea*) by Stone, Wagner and Herbst (in Wagner et al. (1990, 1999), p. 1178). Note: K = Kaua‘i; O = O‘ahu.

**Table:** 

18(15)	Carpels connate 1/6–1/2 their length, sometimes recurved or reflexed before dehiscence; endocarp glabrous or pubescent; leaves rarely inrolled-revolute near base (Megacarpa)	**19**
18	Carpels connate 2/3 to throughout their length, never recurved; endocarp glabrous; leaves often inrolled-revolute near base (Cubicarpa)	**64**
19(18)	Exocarp sparsely to densely puberulent or tomentose, at least towards base along suture	**20**
19	Exocarp glabrous or glabrate, sometimes with a few hairs widely spaced over surface	**49**
49(19)	Endocarp densely and uniformly short-villous; K	***M.cruciata***
49	Endocarp glabrous or sparsely puberulent, especially along suture	**50**
50(49)	Leaves ternate; O	***M.lydgatei***
50	Leaves opposite	**51**
51(50)	Most petioles 0–10 mm long	**52**
51	Most petioles over 10 mm long	**58**
58(51)	Ovary sparsely to densely puberulent or tomentulose, exocarp glabrate to minutely puberulent	**59**
58	Ovary and exocarp glabrous	**60**
60(58)	Inflorescence ramiflorous and axillary, leaves glabrate to pilose-pubescent abaxially, seeds 3–3.5 mm long; K	***M.iolensis***
60	Inflorescence axillary, leaves glabrous abaxially, seeds 4–8 mm long	**60a**
60a(60)	Carpels slightly ascending in fruit, 7–12(–14) mm long, sprawling, prostrate or erect shrubs 1–2 m tall, leaves 2–8(–14) cm long; K	***M.feddei***
60a	Carpels spreading at 180° or reflexed in fruit, 10–24 mm long, sprawling shrubs or trees 1–10 m tall, leaves usually more than 8 cm long	**61**

### ﻿Preliminary conservation assessment. IUCN Red List Category

*Melicopeiolensis* falls into the Critically Endangered (CR) category according to the criteria (B1ab(iii)+B2ab(iii) which reflects a severely limited EOO of 1 km^2^ and AOO of 1 km^2^, a severely fragmented population of only one small subpopulation consisting of 15 mature plants and a continued decline in quality of habitat inferred. The continued decline in quality of habitat for *M.iolensis* is evidenced by severe habitat degradation from invasive non-native mammals such as goats (*Caprahircus* L.), pigs (*Susscrofa* L.) and rats (*Rattus* spp.), along with introduced slugs, insects and disease. In January 2024, we observed the destruction of numerous rare *Cyanea* species in the immediate area by wild goats.

Other serious threats to the habitat include hurricane force winds, flash floods and landslides triggered after torrential rains. Specific invasive non-native plants that displace naturally occurring ones locally include *Erigeronkarvinskianus* DC., (Asteraceae); *Buddleiaasiatica* Lour. (Buddlejaceae); *Sphaeropteriscooperi* (Hook. ex F. Muell.) R.M.Tryon (Cyatheaceae); *Juncusplanifolius* R.Br. (Juncaceae); *Miconiacrenata* (Vahl.) Michelang. (Melastomataceae); *Psidiumcattleyanum* Sabine (Myrtaceae); *Axonopusfissifolius* (Raddi) Kuhlm., *Paspalumurvillei* Steud., *Paspalumconjugatum* P.J.Bergius (Poaceae); and *Rubusrosifolius* Sm. (Rosaceae).

Seeds of *Melicopeiolensis* have been collected by NTBG Science staff and plants are now being cultivated at the NTBG Horticultural Center, Kaua‘i, Hawai‘i.

## Supplementary Material

XML Treatment for Melicope iolensis

## ﻿Additional information

### Author ORCIDs

Kenneth R. Wood https://orcid.org/0000-0001-6446-1154

David H. Lorence https://orcid.org/0000-0002-6735-9531

Warren L. Wagner https://orcid.org/0000-0001-5012-8422

Marc S. Appelhans https://orcid.org/0000-0003-4864-5003

### Data availability

All of the data that support the findings of this study are available in the main text.
